# Genetic Analysis of a Cohort of 275 Patients with Hyper-IgE Syndromes and/or Chronic Mucocutaneous Candidiasis

**DOI:** 10.1007/s10875-021-01086-4

**Published:** 2021-08-14

**Authors:** Natalie Frede, Jessica Rojas-Restrepo, Andrés Caballero Garcia de Oteyza, Mary Buchta, Katrin Hübscher, Laura Gámez-Díaz, Michele Proietti, Shiva Saghafi, Zahra Chavoshzadeh, Pere Soler-Palacin, Nermeen Galal, Mehdi Adeli, Juan Carlos Aldave-Becerra, Moudjahed Saleh Al-Ddafari, Ömür Ardenyz, T. Prescott Atkinson, Fulya Bektas Kut, Fatih Çelmeli, Helen Rees, Sara S. Kilic, Ilija Kirovski, Christoph Klein, Robin Kobbe, Anne-Sophie Korganow, Desa Lilic, Peter Lunt, Niten Makwana, Ayse Metin, Tuba Turul Özgür, Ayse Akman Karakas, Suranjith Seneviratne, Roya Sherkat, Ana Berta Sousa, Ekrem Unal, Turkan Patiroglu, Volker Wahn, Horst von Bernuth, Margo Whiteford, Rainer Doffinger, Zineb Jouhadi, Bodo Grimbacher

**Affiliations:** 1 Center for Chronic Immunodeficiency, Medical Center, Faculty of Medicine, University of Freiburg, Freiburg, Germany; 2 Department of Rheumatology and Clinical Immunology, Medical Center, Faculty of Medicine, University of Freiburg, Freiburg, Germany; 3 Institute for Immunodeficiency, Medical Center, Faculty of Medicine, University of Freiburg, Freiburg, Germany; 4 Immunology Asthma and Allergy Research Institute Tehran University of Medical Sciences , Tehran, Iran; 5 Pediatric Infectious Research Center, Mofid Children Hospital, Shahid Beheshti University of Medical Sciences, Tehran, Iran; 6 Pediatric Infectious Diseases and Immunodeficiencies Unit, Hospital Universitari Vall D’Hebron, Barcelona, Catalonia Spain; 7 Department of Pediatrics, Faculty of Medicine, Cairo University, Cairo, Egypt; 8 Sidra Medicine, Weill Cornell Medicine, Hamad Medical Corporation, Doha, Qatar; 9 Allergy and Immunology Division, Hospital Nacional Edgardo Rebagliati Martins, Lima, Peru; 10 Laboratory of Applied Molecular Biology and Immunology, University of Abou-Bekr Belkaïd, Tlemcen, Algeria; 11 Division of Allergy and Immunology, Department of Internal Medicine, Faculty of Medicine, Ege University, Izmir, Turkey; 12 Division of Pediatric Allergy & Immunology, University of Alabama At Birmingham, Birmingham, AL USA; 13 Departmant of Pediatrics, Division of Pediatric Immunology and Allergy, Faculty of Medicine, Erciyes University, Kayseri, Turkey; 14 Antalya Education and Research Hospital Department of Pediatric Immunology and Allergy, Antalya, Turkey; 15 Bristol Royal Hospital for Children, University Hospitals Bristol NHS Foundation Trust, Bristol, UK; 16 Faculty of Medicine, Uludag University, Bursa, Turkey; 17 Medical Faculty Skopje, 50 Divizija BB, 1000 Skopje, Macedonia; 18 Department of Pediatrics, Dr. Von Hauner Children’s Hospital, University Hospital, LMU Munich, Munich, Germany; 19 First Department of Medicine, Division of Infectious Diseases, University Medical Center , Hamburg-Eppendorf, Germany; 20 UFR Médecine, Université de Strasbourg, Strasbourg, France; 21 Institute of Cellular Medicine, Newcastle University, Newcastle-upon-Tyne, UK; 22 Centre for Academic Child Health, University of Bristol, Bristol, UK; 23 Department of Pediatrics, Sandwell and West, Birmingham Hospitals NHS Trust, Birmingham, UK; 24 Department of Pediatric Allergy and Immunology, Ankara Children’s Hematology Oncology Training and Research Hospital, Ankara, Turkey; 25 Department of Pediatrics, Division of Immunology, Akdeniz University Medical Faculty, Antalya, Turkey; 26 Department of Dermatology and Venerology, Akdeniz University Medical Faculty, Antalya, Turkey; 27 Institute of Immunity and Transplantation, Royal Free Hospital and University College London, London, UK; 28 Acquired Immunodeficiency Research Center, Isfahan University of Medical Sciences, Isfahan, Iran; 29 Serviço de Genética, Hospital de Santa Maria, Centro Hospitalar Universitário Lisboa Norte, and Laboratório de Imunologia Básica, Faculdade de Medicina de Lisboa, Universidade de Lisboa, Lisboa, Portugal; 30 Department of Pediatrics, Division of Pediatric Hematology and Oncology, Faculty of Medicine, Erciyes University, 38010 Melikgazi Kayseri, Turkey; 31 Deparment of Molecular Biology and Genetics, Gevher Nesibe Genom and Stem Cell Institution, GENKOK Genome and Stem Cell Center, Erciyes University, 38010 Melikgazi Kayseri, Turkey; 32 Department of Pediatrics, Division of Pediatric Immunology, Faculty of Medicine, Erciyes University, 38010 Melikgazi Kayseri, Turkey; 33 Department of Pediatric Respiratory Medicine, Immunology and Critical Care Medicine, Charité - Universitätsmedizin Berlin, Freie Universität Berlin, Humboldt-Universität Zu Berlin, and Berlin Institute of Health, Berlin, Germany; 34 Department of Immunology, Labor Berlin GmbH, Berlin, Germany; 35 Charité - Universitätsmedizin Berlin, corporate member of Freie Universität Berlin, Humboldt-Universität Zu Berlin, Berlin Institute of Health (BIH), Berlin-Brandenburg Center for Regenerative Therapies (BCRT), Berlin, Germany; 36 Berlin Institute of Health (BIH), Berlin, Germany; 37 Department of Clinical Genetics, Queen Elizabeth University Hospital, Glasgow, G51 4TF UK; 38 Department of Clinical Biochemistry and Immunology, Addenbrooke’s Hospital, Cambridge, UK; 39 Department of Pediatric Infectious Diseases, Children’s Hospital CHU Ibn Rochd, University Hassan 2, Casablanca, Morocco; 40 German Center for Infection Research (DZIF), Satellite Center Freiburg, Freiburg, Germany; 41 CIBSS – Centre for Integrative Biological Signalling Studies, Albert-Ludwigs University, Freiburg, Germany; 42 RESIST – Cluster of Excellence 2155 to Hanover Medical School, Satellite Center Freiburg, Freiburg, Germany; 43 CCI-Center for Chronic Immunodeficiency, Universitätsklinikum Freiburg, Breisacher Straße 115, 79106 Freiburg, Germany

**Keywords:** Primary immunodeficiency, Hyper-IgE syndrome, Chronic mucocutaneous candidiasis, Genetics, Targeted panel sequencing, Next-generation sequencing

## Abstract

Hyper-IgE syndromes and chronic mucocutaneous candidiasis constitute rare primary immunodeficiency syndromes with an overlapping clinical phenotype. In recent years, a growing number of underlying genetic defects have been identified. To characterize the underlying genetic defects in a large international cohort of 275 patients, of whom 211 had been clinically diagnosed with hyper-IgE syndrome and 64 with chronic mucocutaneous candidiasis, targeted panel sequencing was performed, relying on Agilent HaloPlex and Illumina MiSeq technologies. The targeted panel sequencing approach allowed us to identify 87 (32 novel and 55 previously described) mutations in 78 patients, which generated a diagnostic success rate of 28.4%. Specifically, mutations in *DOCK8* (26 patients), *STAT3* (21), *STAT1* (15), *CARD9* (6), *AIRE* (3), *IL17RA* (2), *SPINK5* (3), *ZNF341* (2), *CARMIL2*/*RLTPR* (1), *IL12RB1* (1), and *WAS* (1) have been detected. The most common clinical findings in this cohort were elevated IgE (81.5%), eczema (71.7%), and eosinophilia (62.9%). Regarding infections, 54.7% of patients had a history of radiologically proven pneumonia, and 28.3% have had other serious infections. History of fungal infection was noted in 53% of cases and skin abscesses in 52.9%. Skeletal or dental abnormalities were observed in 46.2% of patients with a characteristic face being the most commonly reported feature (23.1%), followed by retained primary teeth in 18.9% of patients. Targeted panel sequencing provides a cost-effective first-line genetic screening method which allows for the identification of mutations also in patients with atypical clinical presentations and should be routinely implemented in referral centers.

**Supplementary Information:**

The online version contains supplementary material available at 10.1007/s10875-021-01086-4.

## Introduction

Hyper-IgE syndromes (HIES) and chronic mucocutaneous candidiasis (CMC) constitute rare primary immunodeficiency syndromes with overlapping phenotypes. HIES have traditionally been characterized by the clinical triad of recurrent pneumonias, recurrent skin abscesses, and markedly elevated serum IgE levels [1–3]. Eczema and eosinophilia represent further hallmarks. The most common underlying genetic defects are loss of function mutations of the transcription factor *STAT3* [4, 5], which, in addition to the triad already mentioned, are associated with dental, skeletal, and connective tissue abnormalities [4, 5]. In contrast, *DOCK8* deficiency is characterized by severe viral infections, e.g., with herpes viruses, papilloma viruses, and molluscum contagiosum virus as well as the increased occurrence of malignancies, especially hematological and epithelial cancers. *DOCK8* deficiency is classified as a combined immunodeficiency in the latest International Union of Immunological Societies (IUIS) classification [6, 7]. More recently, mutations in *CARD11*, *ERBB2IP*, *IL6R*, *IL6ST*, *PGM3*, *TGFBR1/2*, and *ZNF431* have been recognized as further genetic causes of HIES-like phenotypes [8–11]. A similar phenotype may be produced by genetic skin disorders such as Comèl–Netherton syndrome, an ichthyosis syndrome caused by mutations in *SPINK5* encoding, a serine protease essential for skin barrier integrity. Also, severe atopic dermatitis (AD) may lead to a similar phenotype, which may be hard to distinguish from primary immunodeficiencies as the impaired barrier function in AD may lead to infections. The hyper-IgE phenotype overlaps with the one of chronic mucocutaneous candidiasis (CMC) in that patients may show increased susceptibility to fungal infections. CMC is characterized by enhanced susceptibility to infections caused by *Candida ssp.* and dermatophytes [12]. While the majority of patients suffer from recurrent skin, nail, or mucous membrane infections, a smaller subgroup of patients develops invasive fungal disease associated with a high burden of morbidity and mortality. In recent years, a growing number of genetic defects underlying CMC have been identified, which confirm a role for both the innate and the adaptive immune systems (*CARD9*, *STAT1*, *ACT1*, *IL-17F*, *IL-17RA*, *IL17RC*, *AIRE*, *IL12B*, *IL12RB1*, *RORC*) in antifungal immunity [13–16]. Depending on the underlying molecular defect, additional clinical manifestations may include autoimmunity, endocrinopathy, increased susceptibility to bacterial infections, and malignancies. Laboratory findings may also include elevated IgE and eosinophilia, adding to a common hyper-IgE phenotype.

With a rising number of known underlying genetic defects and overlapping clinical phenotypes, novel sequencing techniques have been gaining importance to reach a definite diagnosis. Obtaining a genetic diagnosis in these patients with inborn errors of immunity is crucial for identifying the best course of treatment and counseling patients and their families regarding the prognosis and further family planning. The advent of next-generation sequencing (NGS) has since greatly facilitated this process at reduced costs and a shorter turnaround time by allowing the simultaneous analysis of a multitude of genes. In order to promote the time- and cost-effective identification of the genetic diagnosis, we have established a targeted panel sequencing approach relying on Agilent HaloPlex and Illumina MiSeq technologies. Here, we present our results on targeted panel sequencing of known disease-causing genes in a cohort of 275 patients with a clinical diagnosis of HIES or CMC. This approach allowed us to identify 87 mutations in 78 patients.

## Methods

### Patients

This study was conducted under the ethics protocols 239/99–120,733 and 302/13 (ethics committee of the University Hospital of Freiburg, Germany). All patients, or for children their legal guardians, have consented according to local ethics guidelines. DNA or whole blood samples of patients who had either a clinical diagnosis of HIES or CMC, and whose samples were referred to our laboratory for genetic workup on a research basis by their local physicians, were obtained. Around 211 patients had a clinical diagnosis of HIES, while 64 patients were diagnosed with CMC. With two exceptions, all patients were unrelated index patients. If DNA samples were available, other family members were subsequently investigated for the detected mutations by Sanger sequencing. The investigated patients were from Algeria (5 patients), Belgium (2), Bosnia (1), Brazil (1), Canada (2), Chile (2), Colombia (2), Denmark (1), Egypt (30), Finland (1), France (3), Germany (41), Great Britain (44), Greece (3), India (2), Iran (34), Ireland (8), Israel (5), Italy (10), Lebanon (1), Libya (2), Macedonia (2), Malaysia (1), Morocco (21), Oman (1), Peru (3), Portugal (3), Qatar (1), Slovakia (1), Spain (3), Switzerland (2), Tunisia (4), Turkey (29), USA (3), and the West Indies (1). The cohort comprises an initial group of 90 patients, in whom conventional diagnostics including Sanger sequencing of the suspected underlying target gene (*STAT3*, *DOCK8*, *PGM3*, *STAT1*) had not led to a definite molecular diagnosis. Subsequently, the cohort was expanded to include patients with a clinical diagnosis of HIES or CMC without prior Sanger sequencing.

### DNA Extraction

DNA extraction was performed according to local protocols. In brief, erythrocytes were lysed with RBC buffer, and the remaining nucleated cells were subjected to Qiagen Cell Lysis Solution. Qiagen Protein Precipitation Solution was used to precipitate the proteins. The DNA was subsequently precipitated with isopropanol, washed with ethanol, and resuspended and stored in Qiagen DNA Hydration Solution.

### Panel Design

A customized gene panel was designed through Agilent’s web-based SureDesign application. The panel design initially comprised 15 genes in which published evidence has established that mutations can cause HIES or CMC and was updated regularly to optimize coverage and include new candidate genes. A list of sequenced gene sets is found in Supplemental Table 1.

### Target Enrichment and Sequencing

Targeted enrichment was performed with Agilent’s HaloPlex Target Enrichment System for Illumina sequencing. The manufacturer’s instructions as detailed in Agilent’s user manual were followed. In brief, DNA samples were subjected to digestion by adding a restriction enzyme master mix prepared following the kit’s protocol and an incubation step at 37 °C, and digestion was validated by gel electrophoresis. Subsequently, the restriction fragments were hybridized to the HaloPlex probe capture library by the addition of a hybridization master mix and indexing primer cassettes. The mix was incubated at 95 °C for 10 min followed by a 3-h incubation at 54 °C. The target DNA was captured with a biotin–streptavidin system using HaloPlex Magnetic Beads. After a washing step, the circular fragments were closed through a ligation reaction at 55°. The captured target libraries were amplified by PCR with the master mix prepared according to the manufacturer’s instructions. In a final step, the amplified target libraries were purified through AMPure XP beads and washed in ethanol. Enrichment was validated on an Agilent TapeStation system.

Samples were pooled in equimolar amounts for multiplex sequencing on an Illumina MiSeq system. An Illumina v2 reagent kit was used, and the provided protocol was followed. Libraries were denatured with NaOH and diluted to a final concentration of 8–12 pM. For loading and starting the MiSeq system, the manufacturer’s instructions as detailed in the MiSeq System User Guide were followed.

### Data Analysis

Data analysis was performed using Agilent’s SureCall software. In brief, this included the trimming of adapter sequences and alignment to the human reference genome (hg19/GRCh37) using Burrows–Wheeler aligner. The Agilent SNP caller was used for detecting single nucleotide variants. SNP filtering, mutation classification, and annotation were performed as part of the SureCall analysis workflow. Copy number variants were determined from sequencing coverage data using a genomic analysis toolkit. Single nucleotide variants with an allele frequency > 0.01 in the general population were excluded. Detected missense, nonsense, frameshift, and splice site mutations were annotated. For prioritization databases, such as HGMD (Human Gene Mutation Database) [17], ExAc (Exome Aggregation Consortium) [18], and dbSNP (the Single Nucleotide Polymorphism Database) [19], as well as polymorphism phenotyping tools/mutation severity predictors such as PolyPhen2 [20] and combined annotation-dependent depletion (CADD) [21] were used.

### Quality Control Data

The average read depth of all detected mutations was 914 × . Mutations were confirmed by Sanger sequencing or PCR amplification in the case of larger deletions. Around 91.8% of all bases in target regions were covered at least 100-fold, and 97.6% were covered at least 20-fold.

### Sanger Sequencing

All detected mutations were confirmed by Sanger sequencing according to established protocols [22]. Regions of interest were amplified from genomic DNA by standard PCR. PCR primers were used for Sanger sequencing according to standard techniques. All primer sequences are available upon request. Sequencing was performed on an AB3130xl Genetic Analyzer, or PCR products were sent for commercial sequencing at GATC (Konstanz, Germany).

### PCR

In order to confirm suspected copy number variations (deletions), the regions in question were amplified from genomic DNA by PCR as detailed previously. Amplification of patient and control DNA was checked by agarose gel electrophoresis.

### Flow Cytometry

If available, functional testing on patient cells was performed for novel variants. While not diagnostic, phosphorylation assays contribute to the assessment of potential pathogenicity and mechanism of STAT variants. Cell surface staining or intracellular staining for the assessment of variants was performed by flow cytometry. PBMCs from patients were stained with pPSTAT3-PE, pSTAT1-Alexa 647, or IL17RA-APC and were subsequently analyzed on a BD FACS Canto II.

## Results

**Fig. 1:**
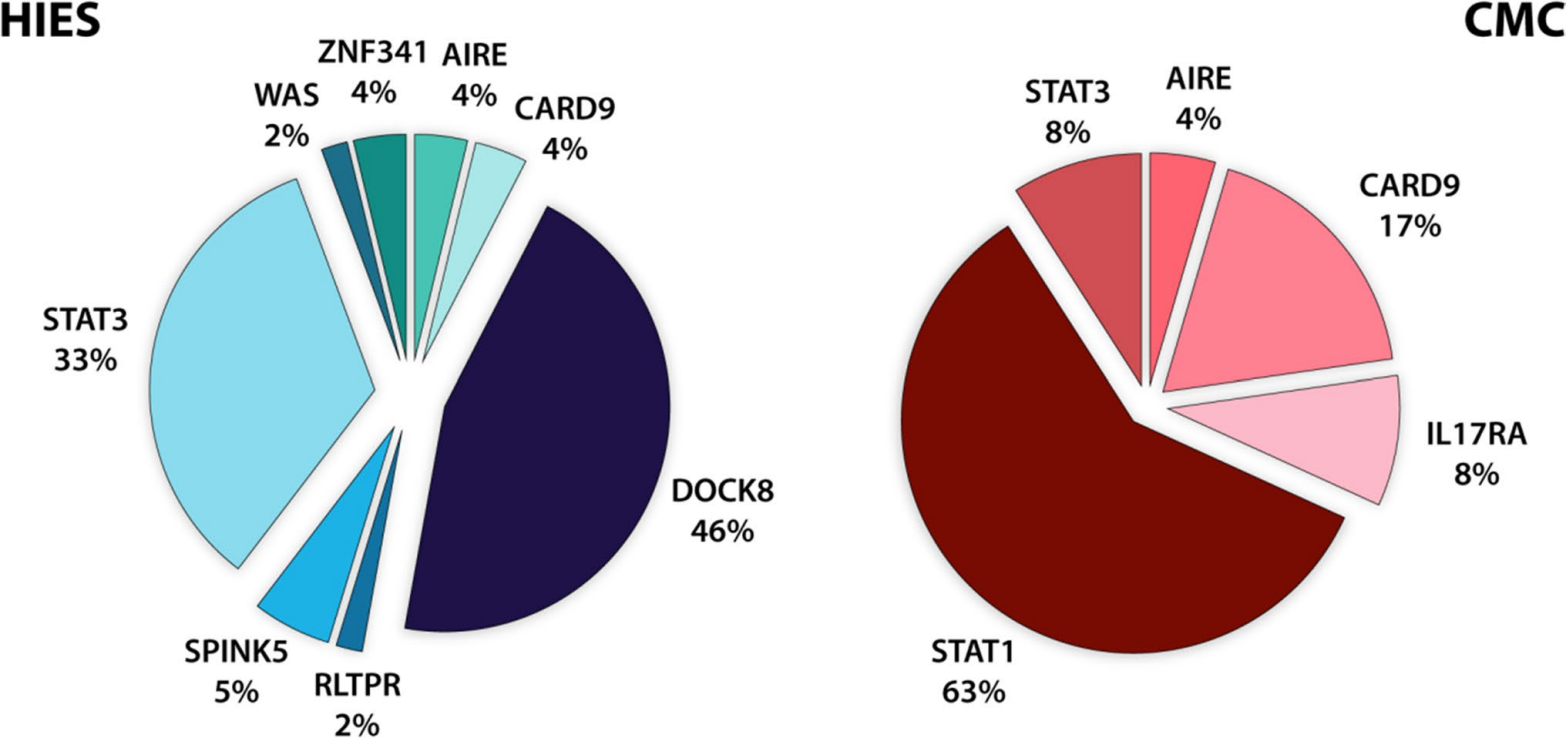
Frequency of genetic defects in patients with a clinical diagnosis of HIES and CMC: In patients with a clinical diagnosis of HIES, *DOCK8* and *STAT3* mutations constituted the most common defects, whereas in CMC patients, *STAT1* mutations were the most common defects

Within this study, we analyzed a cohort of 275 patients by targeted panel sequencing, out of whom 211 had been clinically diagnosed with HIES by their referring physicians and 64 with CMC. Targeted panel sequencing identified underlying disease-causing mutations in 78 patients, i.e., 28.4% of all analyzed patients. Specifically, we detected 25 homozygous and one compound heterozygous mutations in *DOCK8*, 21 heterozygous *STAT3* mutations, 13 heterozygous mutations in *STAT1*, five homozygous and one compound heterozygous mutation in *CARD9*, two compound heterozygous and one heterozygous mutation in *AIRE*, three homozygous mutations in *SPINK5*, one homozygous and one compound heterozygous mutation in *IL17RA*, two homozygous *ZNF341* mutations, one homozygous *RLTPR* mutation, one homozygous mutation in *IL12RB1*, and one hemizygous *WAS* mutation in this cohort (Fig. 1). The average coverage of detected mutations in this study amounted to 914 reads.

The mean age of patients at the time point of sequencing was 17.9 years (range: 1–62 years; mean for patients with clinical hyper-IgE phenotype: 15.7 years, for patients with CMC: 24.3 years). Around 55.1% of analyzed patients were male and 44.9% female. Around 20.5% were reported to be consanguineous. The mean age at first clinical presentation was 8.41 years (median: 5 years, range: 0–51 years).

**Fig. 2:**
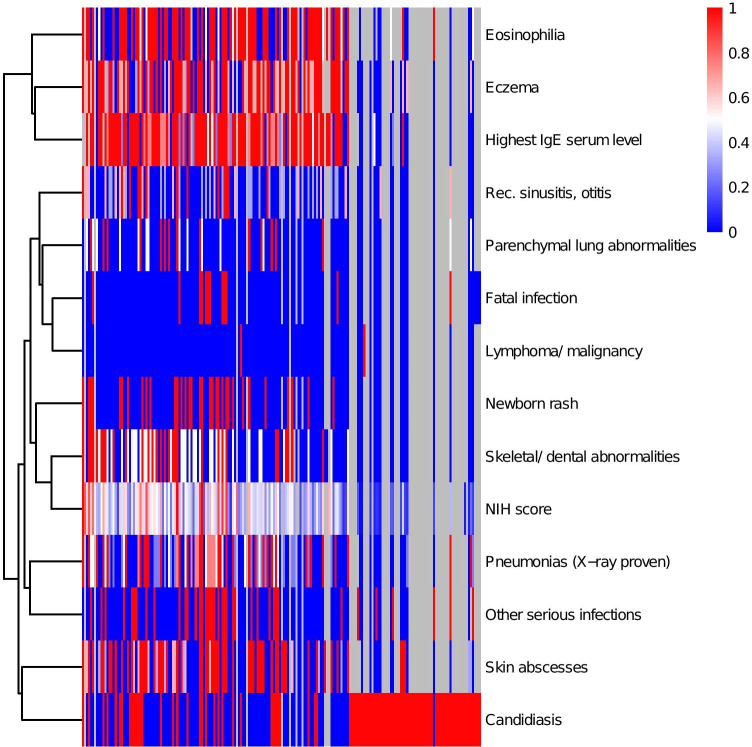
Clinical patient characteristics: heat map showing relative scoring for presence and severity of clinical patient characteristics in this cohort. Eosinophilia, elevated IgE, and eczema cluster together

Clinical data were available for 183 patients and are summarized in Fig. 2. The majority of patients had elevated IgE levels (81.5%), and 62.9% had eosinophilia. The most commonly reported symptom was eczema (71.7%). Regarding infections, 54.7% of patients had a history of radiologically proven pneumonia, while 28.3% of patients were reported to have had other serious infections. Twelve patients had passed away from infectious causes. Around 53% of patients in this cohort had a history of mucocutaneous candidiasis or fungal infection and 52.9% had a history of skin abscesses. About 37.2% had recurrent sinusitis with at least 3 episodes per year. Around 46.2% of patients had skeletal or dental abnormalities with a characteristic face being the most commonly reported item (23.1%), followed by retained primary teeth reported in 18.9% of patients. Around 9.8% had hyperextensibility and 8.5% a history of fractures with inadequate trauma.

Sixteen patients were reported to have enteropathy. Neurologic features were generally rare (reported in 7 patients) and included epilepsy, delayed development, encephalomalacia, and cerebral atrophy. Malignancy or suspicion thereof was only reported in three patients, one of which had a sarcoma, one multiple skin tumors, and another a lymphoma. HIES patients had an average NIH HIES score of 34.0 points (*n* = 122; min.: 6, max.: 73), whereas CMC patients scored an average of 9.75 points (*n* = 20).

### Identified Genetic Defects in Patients with a Clinical Diagnosis of HIES

Out of 211 patients referred for genetic workup with a clinical diagnosis of HIES, we were able to make a genetic diagnosis in 54 patients, i.e., 25.6%. A detailed list of detected mutations and clinical presentations of patients can be found in Table 1. In this cohort, *DOCK8* mutations were the most common underlying genetic defect. A total of 26 patients were shown to have homozygous or compound heterozygous *DOCK8* mutations*.* Out of these, four patients had splice site defects, six nonsense mutations, and five frameshift mutations. One patient had a one amino acid deletion, whereas another patient (HIES53) had a compound heterozygous mutation consisting of a heterozygous one amino acid deletion and a heterozygous splice site variant. Staining for *DOCK8* protein confirmed severely reduced protein expression by flow cytometry. Seven patients were found to have larger homozygous exonic deletions, which could be confirmed by PCR amplification.HIES46

**Table 1:** Detected mutations in HIES patients: summary of detected disease-causing variants and clinical presentation of patients. AA, amino acid; hom, homozygous; het, heterozygous; hemi, hemizygous

Patient	From	NIH HIES score	Age at first clin. presentation (years)	Age at genet. diagn. (years)	Gene	Base exchange	AA exchange	Zygosity	Variant published? (ref.) Functional validation?	Clinical presentation
HIES01	Morocco	35	10	16	*AIRE*	c.755C > T	p.P252L	het	Yes [43] unclear	Asthma, sinusitis, mild eczema, marfanoid habitus, osteoporosis with a history of multiple fractures upon inadequate trauma, multinodular goiter, pulmonary cyst, elevated IgE, no consanguinity
					*AIRE*	c.91delG	p.V31Sfs*150	het	No	
HIES02	Egypt	33	1	4	AIRE *	c.91delG	p.V31Sfs*150	het	No	Recurrent pneumonias, severe sinusitis, eczema with recurrent skin infections, diarrhea requiring hospitalization, consanguinity. *Also has *DOCK8* mutation
					AIRE *	c.755C > T	p.P252L	het	Yes [43] unclear	
HIES03	Turkey	40	5	17	*CARD9*	c.883C > T	p.Q295*	hom	Yes [15] Yes	Clinical diagnosis of hyper-IgE syndrome due to IgE levels > 20,000 IU/l and marked eosinophilia (HIES score of 40 points). Candida meningitis
HIES04	Iran	23	7	7	*CARD9*	c.1118C > G	p.R373P	hom	Yes [44] Yes	Aphthous stomatitis, persistent thrush in infancy, candidal nail infection in early childhood, hearing loss, hypereosinophilia, elevated IgE, retroperitoneal lymphadenopathy, abdominal lymph node biopsy with proof of necrotizing granulomatous inflammation, PAS staining positive for hyphae
HIES05	Egypt	23	3	4	*DOCK8*	c.5962-1G > A	IVS45-1(G > A)	hom	No	Eczema, hepatosplenomegaly, pneumonias, severe persistent diarrhea, CD4 lymphopenia, elevated IgE, arrest of B cell maturation with very low CD27 expression. Consanguinity
HIES06	Oman	32	9	15	*DOCK8*	c.3787delA	I1263*	hom	No	Recurrent sinusitis with bilateral mastoiditis, severe fungal nail bed infections with bacterial superinfection, headache, diplegia, choreiform and dystonic movements
HIES07	Iran	47	13	15	*DOCK8*	c.5656_5657delAA	p.K1886Efs*10	hom	No	Recurrent pneumonias, sinusitis, newborn rash, eosinophilia
HIES08	Turkey	8	8	8	*DOCK8*	c.2864_2865 insAT	p.V956Lfs*13	hom	No	Disseminated verrucosis affecting especially the hands. Normal IgE levels, eosinophilia of 30%. Consanguinity, sister passed away prior to genetic confirmation of HIES in our patient but also had marked hypereosinophilia
HIES09	Great Britain	n.a	14	-	*DOCK8*	c.5491-2A > G	IVS42-2(A > G)	hom	No	Diffuse large B-cell lymphoma, achieved remission, but developed methotrexate encephalopathy under treatment with significant neurological sequelae, passed away due to chest infection
HIES10	Egypt	29	3	6	*DOCK8*	c.5132C > A	p.S1711*	hom	Yes [45] Yes	Eczema, pneumonia. Consanguinity, 2 siblings deceased (brother with neonatal sepsis, sister deceased age 3 with similar phenotype)
HIES11	Egypt	20	< 1	2	*DOCK8*	c.2959_2961delTTC	p.F986del	hom	No	Pneumonia, chronic suppurative otitis, axillary abscesses, newborn rash, mucocutaneous candidiasis, severe herpetic infection, splenomegaly, eosinophilia, elevated IgE (16,390 IU/ml), consanguinity, 2 siblings deceased aged 4 and 6y
HIES02	Egypt	33	1	4	DOCK8*	c.3135delT	p.F1045Lfs*2	hom	Yes [46] No	Recurrent pneumonias, severe sinusitis, eczema with recurrent skin infections, diarrhea requiring hospitalization, consanguinity, 1 affected brother with same *DOCK8* mutation. *Also has *AIRE* mutation
HIES12	Egypt	22	3	4	*DOCK8*	c.3135delT	p.F1045Lfs*2	hom	Yes [46] No	Neonatal seizures, progressive eczema w superinfection, otitis, septic arthritis of left hip, consanguinity, 1 affected sibling
HIES13	Iran	66	< 1	9	*DOCK8*	c.2721_2724delGAGA	p.R908Kfs*15	hom	No	Recurrent pneumonias, recurrent sinusitis, newborn rash, eosinophilia
HIES14	Egypt	15	3	4	*DOCK8*	c.5132C > A	p.S1711*	hom	Yes [45] Yes	Neonatal rash, ubiquitous eczema, T lymphopenia, consanguinity, two affected siblings
HIES15	Egypt	14	5	6	*DOCK8*	c.5132C > A	p.S1711*	hom	Yes [45] Yes	Consanguinity, chronic diarrhea, sinusitis, draining ears, eczema, failure to thrive, eosinophilia, T lymphopenia, elevated IgE
HIES16	Egypt	24	5	7	*DOCK8*	c.5132C > A	p.S1711*	hom	Yes [45] Yes	Bronchial asthma, severe eczema, recurrent ear problems resulting in impairment of hearing requiring hearing aids, cervical lymphadenopathy, biopsy showed reactive follicular hyperplasia, mild hepatomegaly, warts. Elevated IgE. Consanguinity, two sibling deaths
HIES17	Iran	35	n.a	13	*DOCK8*	c.1803 + 1G > C	IVS17 + 1(G >C)	hom	No	Recurrent pneumonias, recurrent skin abscesses, moderate eczema, eosinophilia
HIES18	Egypt	34	5	8	*DOCK8*	c.709G > T	p.E237*	hom	No	Recurrent viral upper respiratory tract infections, severe eczema, recurrent skin and scalp abscesses, severe eczema herpeticum, repeated episodes of diarrhea, onychomycosis, elevated IgE. Consanguinity
HIES19	Egypt	18	3	4	*DOCK8*	c.5132C > A	p.S1711*	hom	Yes [45] Yes	Recurrent rash, asthma, chest infections, history of diarrhea, suspected cholangitis. Elevated IgE. Consanguinity
HIES20	Egypt	42	< 1	4	*DOCK8*	c.4627-1G > C	IVS37-1(G > C)	hom	No	Transient respiratory distress with NICU admission after birth, neonatal rash in napkin area, facial eczema, severe persistent diarrhea since age of 3 months, bilateral draining ears, pneumonia, recurrent scalp abscesses, eosinophilia, marked IgE elevation. Consanguinity, one sibling died with respiratory distress on first day of life after preterm birth
HIES21	Egypt	22	5	8	*DOCK8*	c.709G > T	p.E237*	hom	No	Developed severe oral ulcers and facial eczema with impetiginous lesions aged 2, followed by severe persistent diarrhea, which never resolved, bilateral draining ears, severe eczema, superficial scalp and skin abscesses, onychomycosis. Elevated IgE, eosinophilia. Consanguinity, cousin of HIES18
HIES22	Iran	35	4	7	*DOCK8*	n.a	Ex1-26 deletion	hom	Yes [47] Yes	Recurrent infections, candidiasis, BCG lymphadenitis, chronic diarrhea, severe atopy, elevated IgE. Aortic coarctation. Consanguinity
HIES23	Egypt	24	5	10	*DOCK8*	n.a	Ex26-33 deletion	hom	No	Skin rash, recurrent eczema, persistent diarrhea; at age 3 car accident w skull fissure and one episode of convulsions, cCT normal. Failure to thrive. At age 9 pneumonitis with ground glass appearance, hepatomegaly, ascites, cCT w evidence of cortical brain atrophy, hypoplastic pituitary gland; age 10 episode of disturbed consciousness and intractable convulsions, suspicion of CNS vasculitis. Elevated IgE, recurrent eosinophilia. Consanguinity
HIES24	Egypt	30	2	3	*DOCK8*	n.a	Ex6-7 deletion	hom	No	Boils covering the whole scalp since birth, eczema, draining ears, multiple episodes of diarrhea. Elevated IgE, eosinophilia. Consanguinity, two sibling deaths
HIES25	Iran	17	11	11	*DOCK8*	n.a	Ex 25–26 deletion	hom	Yes [48]	11-year old boy with recurrent ulcerovegetative genital lesion (biopsy: pyoderma vegetans), itchy macular lesions on the palms and soles, periocular warty lesions. Recurrent airway infections. Improvement under aciclovir, IVIG and methylprednisolone pulse therapy. Elevated IgE, consanguinity
HIES26	Portugal	22	6	7	*DOCK8*	n.a	Ex 1–12 deletion	hom	No	Recurrent respiratory and cutaneous infections. Elevated IgE
HIES27	Egypt	35	5	7	*DOCK8*	n.a	Ex1-48 deletion	hom	Yes [47] Yes	Recurrent otitis, 2 × pneumonia, recurrent diarrhea, s/p liver abscess, cholangitic microabscesses, splenomegaly, recurrent scalp abscesses, severe facial and retroauricular eczema. Consanguinity
HIES53	Germany	25	4	41	*DOCK8*	c.4257_4259del	p.K1422del	het	No	Eczema since childhood, atopy with multiple allergies, recurrent pneumonias, generalized warts, recurrent skin tumors, recurrent meningitis (*Hemophilus influenzae*, *S. pneumoniae*), passed away due to JC virus infection. Positive family history
					*DOCK8*	c.1422G > A	p.Q474 = (splice variant)	het	No	
HIES54	Iran	23	n.a	10	*DOCK8*	n.a	Ex11-28 deletion	hom	No	Severe eczema, eosinophilia, IgE elevation, age of onset 2 years. Consanguinity
HIES28	Iran	17	3	4	*RLTPR*/*CARMIL2*	c.467-1G > A	IVS6-1(G > A)	hom	No	Severe dermatitis from infancy on, multiple episodes of sinusitis, nail candidiasis necessitating systemic treatment, multiple allergies, hyperreactive airway disease, history of eosinophilic esophagitis (eosinophilia of 27.1%). Mild IgE elevation, IgG/A/M within normal range, lymphocytosis, decreased FOXP3^+^ CD4^+^ regulatory T cells
HIES29	Qatar	31	< 1	1	*SPINK5*	c.2215C > T	p.Q739*	hom	No	Severe generalized erythroderma including palms and soles. Hypercalcemia, elevated IgE. Consanguinity, two siblings died in infancy
HIES30	Iran	25	2	7	*SPINK5*	c.2259_2259delA	p.N755Mfs*27	hom	Yes [49] No	Atopy, eczema, failure to thrive, recurrent infections, history of atypical mycobacterial infection, recurrent fungal infections. Elevated IgE and immunoglobulins, lymphocyte subclasses within normal ranges. Consanguinity
HIES31	Turkey	32	< 1	6	*SPINK5*	c.2112 + 2 T > A	IVS22 + 2(T > A)	hom	No	3-year-old boy, consanguinity. Treatment-refractory dermatitis since early infancy, nail dystrophy, coarse face. No history of recurrent infections. Elevated IgE, eosinophilia
HIES32	Iran	63	16?	17	*STAT3*	c.1594 A > C	p.K531Q	het	No	Recurrent pneumonias, recurrent skin abscesses, oral candidiasis, retained primary teeth, high palate, characteristic facies
HIES33	Iran	66	n.a	31	*STAT3*	c.995G > A	p.H332Y	het	Yes [50] Yes	Recurrent pneumonias, recurrent skin abscesses, newborn rash, severe eczema, 2 fractures without adequate trauma, systemic candidiasis, fatal infection
HIES34	Iran	31	1	3	*STAT3*	c.1144C > T	p.R382W	het	Yes [4] Yes	Recurrent pneumonias, severe eczema
HIES35	Peru	43	2	4	*STAT3*	c.1145G > A	p.R382Q	het	Yes [4] Yes	Recurrent staphylococcal skin infections, erythematous maculopapular pruritic rash since birth, history of multiple abscesses, pneumonia, history of leukemoid reaction
HIES36	Iran	21	n.a	9	*STAT3*	c.1909G > A	p.V637M	het	Yes [5] Yes	Eczema with vesicular rash, multiple food allergies, history of anaphylactic shock, asthma, recurrent sinusitis, retention of primary teeth. Marked IgE elevation, no consanguinity
HIES37	Morocco	50	2	13	*STAT3*	c.1850G > A	p.G617E	het	Yes [38] No	Atopic dermatitis, cold abscesses, pyoderma, pneumonia, pneumatocele, history of fungal infections of nail and outer ear canal, dental retention, characteristic facies. Elevated IgE, eosinophilia
HIES38	Morocco	17	8	13	*STAT3*	c.2144 + 1G > A	IVS22 + 1(G > A)	het	Yes [51] No	Atopic dermatitis in infancy, history of pneumonia, fungal infection of scalp, molluscum contagiosum, *Microsporum canis* infection, eosinophilia, IgE normal
HIES39	Iran	73	19	23	*STAT3*	c1971_1971delT	p.Y657*	het	Yes [52] No	Recurrent pneumonias, bronchiectasis, recurrent skin abscesses, newborn rash, severe eczema, high palate, scoliosis, fatal infection
HIES40	Germany	41	4	33	*STAT3*	c.2131A > T	p.I711F	het	No	Recurrent pneumonias, arched palate, recurrent skin abscesses, s/p scrotal abscess, candida esophagitis, suspected intraarticular empyema of shoulder
HIES41	Turkey	40	12	19	*STAT3*	c.1387_1389delGTG	p.V462del	het	Yes [4] Yes	Recurrent skin abscesses, moderate eczema, high palate, characteristic facies
HIES42	Great Britain	32	5	20	*STAT3*	c.1591A > G	p.K531E	het	Yes [53] Yes	Recurrent pneumonia, recurrent abscesses, retention of deciduous teeth, mild hyperextensibility of joints, elevated IgE
HIES43	Colom-bia	48	4	8	*STAT3*	c.1395_1397delCAA	p.N466del	het	No	Severe eczema since early infancy, recurrent cutaneous abscesses, several episodes of suppurative otitis media and pneumonias (> 3) that required frequent hospitalizations and topical as well as IV antibiotics, development of pneumatoceles. Elevated IgE
HIES44	Germany	35	3	40	*STAT3*	c.2125A > G	p.K709E	het	Yes [54] Yes	Recurrent pneumonias, s/p abscess forming pneumonia with pneumatocele formation, later pneumonectomy of right lung due to chronic abscess-forming pneumonia, recurrent abscesses, therapy-resistant onychomycosis, verrucosis
HIES45	Turkey	45	1	25	*STAT3*	c.1144C > G	p.R382G	het	Yes [55] No	Recurrent pneumonias with bronchiectasis and pneumatocele formation, s/p resection of right upper lobe, recurrent cutaneous abscesses, cutaneous candida infection, onychomycosis, eczema, pronounced scoliosis with resection of multiple ribs, trigeminal neuralgia after extirpation of infected branchial cyst
HIES46	Germany	36	36	46	*STAT3*	c.1723 T > G	p.Y575D	het	No	Pulmonary abscess formation, s/p pneumonectomy, recurrent abscesses, recurrent oral thrush, onychomycosis, multiple fractures with inadequate trauma
					*STAT3*	c.1711C > T	p.L571F	het	No	
HIES47	Great Britain	n.a	< 1	6	*STAT3*	c.1110-2A > G	IVS11-2(A > G)	het	Yes [51] Yes	Recurrent abscesses, history of liver abscess at the age of 6 months (secondary to *Staphylococcus aureus* with PVL toxin), lung abscess, eczema, Influenza A infection requiring ECMO
HIES48	Egypt	25	1	2	*STAT3*	c.1909G > A	p.V637M	het	Yes [5] Yes	Extensive severe eczema, recurrent chest infections, marked gingivitis, hepatomegaly, *Cryptosporidium* infection. Elevated IgE
HIES49	Macedonia	43	< 1	9	*STAT3*	c.1909G > A	p.V637M	het	Yes [5] Yes	Eczema, dental abnormalities including high arched palate and severe caries, history of multiple bone fractures in early childhood, recurrent lower respiratory tract infections, two giant pneumatoceles in right hemithorax. Elevated IgE, eosinophilia
HIES50	Iran	20	n.a	7	*WAS*	c.1208C > T	p.P403L	hom	Yes [26] No	Eczema, hypereosinophilia (69%) with hypereosinophilic skin lesions, elevated IgE (15,064 IU/ml), normal MPV and platelet count
HIES51	Turkey	22	10	14	*ZNF341*	c.1135C > T	p.R386*	hom	Yes [23] Yes	Mild respiratory tract infections, skin abscesses, severe eczema, micrognathia. This patient was previously published in Frey-Jakobs et al. [23]
HIES52	Turkey	62	n.a	34	*ZNF341*	c.1135C > T	p.R386*	hom	Yes [23] Yes	Severe upper and lower respiratory tract infections, bronchiectasis, skin abscesses, severe eczema, micrognathia. This patient was previously published in Frey-Jakobs et al. [23]

**Fig. 3:**
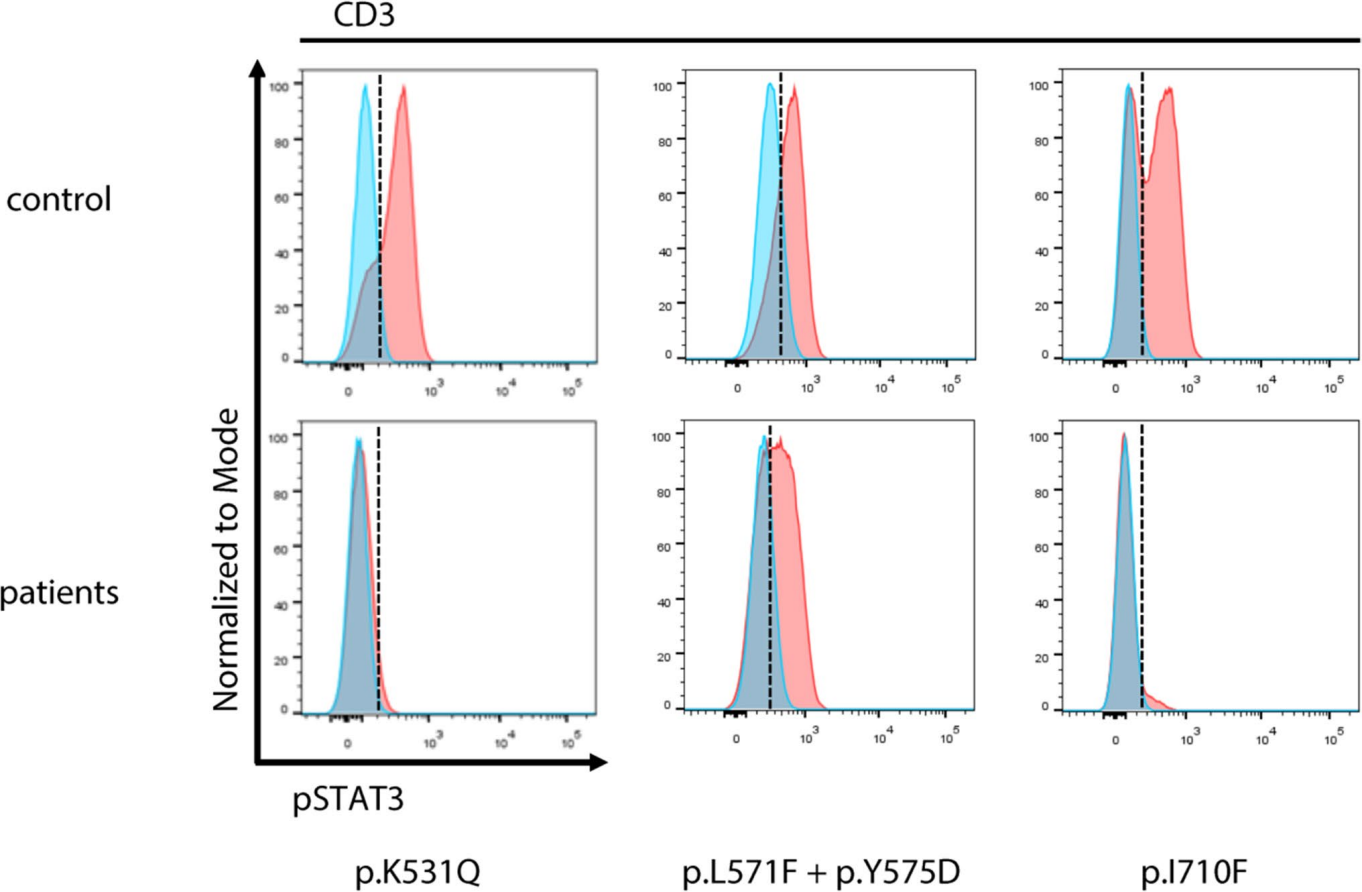
Functional assessment of novel *STAT3* defects: flow cytometric analysis of pSTAT3 in CD3^+^ cells of patients shows hypophosphorylation of *STAT3* upon stimulation with IL-6 for the assessed variants, p.K531Q and p.I710F, and to a lesser extent, p.L571F/p.Y575D. While not diagnostic, this result suggests pathogenicity for the assessed variants

Eighteen patients had heterozygous *STAT3* mutations. Of these, two patients had splice site mutations, one a nonsense mutation, two single amino acid deletions, and 13 patients had missense mutations. Functional testing was performed for the novel variants p.K531Q and p.I711F as well as for patient HIES46, who had two previously undescribed missense variants, p.L571F and p.Y575D. Testing showed reduced *STAT3* phosphorylation consistent with a loss of function for two patients and mildly reduced phosphorylation of unclear significance for the third patient (Fig. 3). Furthermore, we identified two patients with a homozygous *ZNF341* nonsense mutation [23]. Biallelic mutations in *ZNF341* lead to diminished *STAT3* expression, resulting in hyper-IgE syndrome [23, 24]. In addition to recurrent respiratory infections, skin abscesses, and severe eczema, the two patients suffered from micrognathia.

Interestingly, two patients clinically diagnosed with HIES were shown to have previously published *CARD9* mutations (p.R373P and p.Q295*) associated with chronic mucocutaneous candidiasis, respectively, deep dermatophytosis. Patient HIES03 had IgE levels of > 20,000 IU/l and marked eosinophilia adding towards a total NIH HIES score of 40 points [25]. However, he had suffered from candida meningitis, and thus invasive fungal infection, hinting towards a *CARD9* defect. Notably, no clinical sign or history of lung disease — typical of HIES — was present. Patient HIES04 suffered from persistent thrush in infancy and candida nail infection; however, he had been diagnosed with HIES due to elevated IgE and hypereosinophilia.

Three patients clinically diagnosed with HIES had homozygous mutations in *SPINK5*, which cause Comèl–Netherton syndrome, a rare syndrome associated with erythroderma, ichthyosis, short and brittle “bamboo” hair, atopy, and elevated IgE. One patient had a nonsense mutation, one a frameshift, and one a splice site mutation. All patients had severe eczema and elevated IgE, and only one of the three patients did not have a history of recurrent infections.

Notably, another patient (HIES50) clinically diagnosed with HIES was shown to have a hemizygous mutation in *WAS* (p.P403L), which had previously been published as causing mild immunodeficiency, muscle dystonia, and thrombocytopenia [26]. The patient had eczema, marked hypereosinophilia (69%, absolute count: 21,962/µl) with hypereosinophilic skin lesions, and elevated IgE levels (15,064 IU/ml). Platelet count and platelet volume were noted to be normal.

Additionally, we have identified one patient with a homozygous splice site defect in *RLTPR*, who had been clinically diagnosed with HIES. The patient suffered from severe dermatitis from infancy on and, in addition, had multiple episodes of sinusitis and nail candidiasis necessitating systemic treatment as well as hyperreactive airway disease. He has multiple allergies and was diagnosed with eosinophilic esophagitis at the age of 2 years (eosinophilia of 27.1%). The clinical phenotype is thus similar to the one published by Alazami et al. [27], although this patient so far only demonstrated mild signs of immunodeficiency compared to the combined immunodeficiency phenotype described originally by Wang et al. [28]. Recurrent measurements showed mildly elevated IgE, but other immunoglobulins and vaccination responses were normal. Further laboratory workup showed persistent lymphocytosis. The patient was shown to have decreased FOXP3^+^ CD4^+^ regulatory T cells in line with the so far described phenotype.

Furthermore, two patients had compound heterozygous mutations in *AIRE*. Patient HIES01 suffered from asthma, sinusitis, and mild eczema and was found to have a multinodular goiter and a large pulmonary cyst. Laboratory workup showed elevated IgE levels. Additionally, he had a Marfanoid habitus and osteoporosis with a history of multiple fractures upon minor trauma, leading to the clinical diagnosis of HIES. The other patient (HIES02) additionally had a homozygous frameshift mutation of *DOCK8* and thus had presented with recurrent pneumonias, severe sinusitis, and eczema with recurrent skin infections and diarrhea.

### Identified Genetic Defects in Patients with a Clinical Diagnosis of CMC

**Fig. 4:**
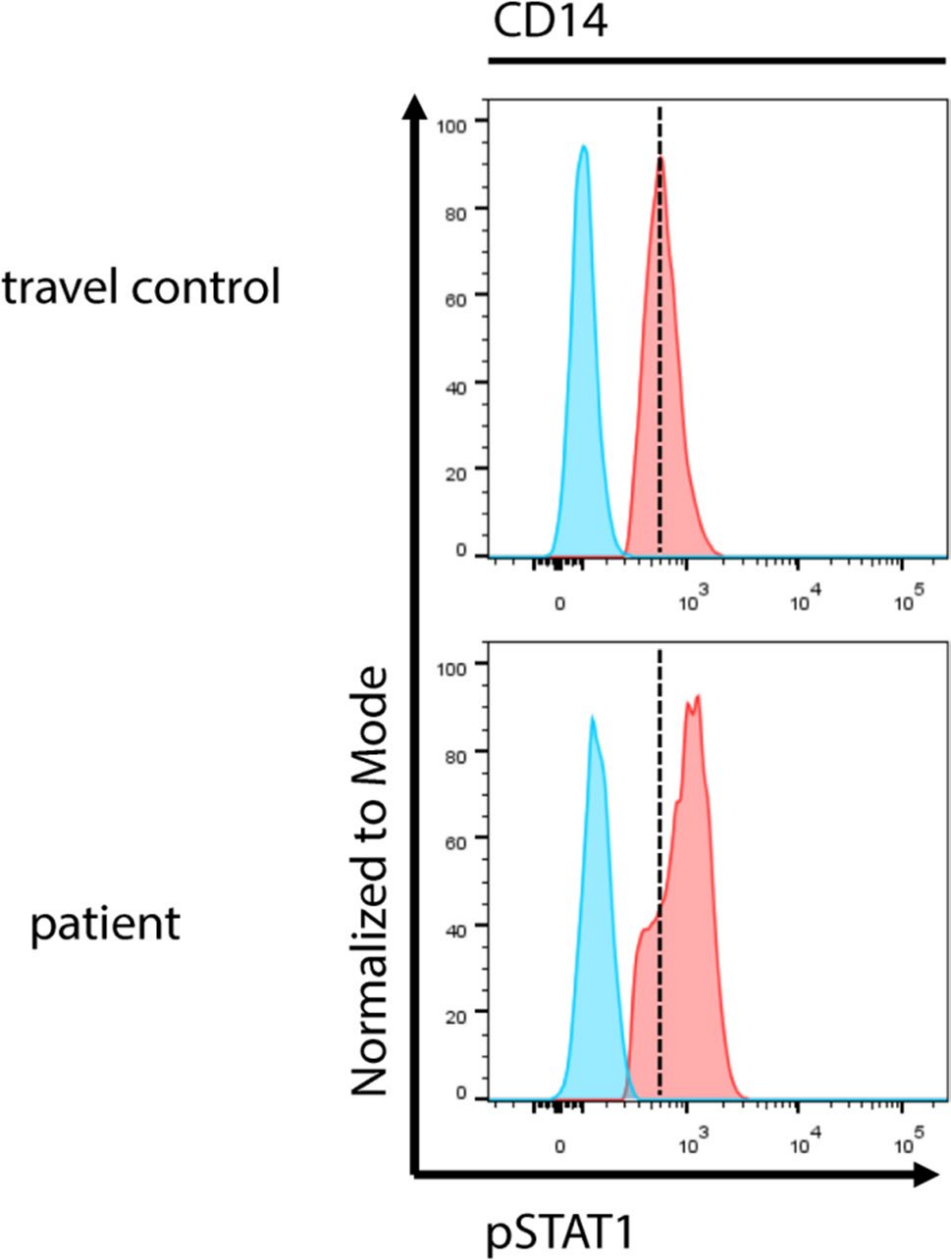
Functional assessment of novel *STAT1* variant p.E282D: flow cytometric analysis of pSTAT1 in CD14^+^ cells of patient CMC07 demonstrates that the novel *STAT1* variant p.E282D leads to hyperphosphorylation of *STAT1* upon stimulation with IFNα, confirming a gain-of-function mutation

Out of 64 patients with a clinical diagnosis of CMC, targeted panel sequencing allowed us to make a genetic diagnosis in 24 patients, i.e., 37.5%. A detailed list of detected mutations and clinical presentations of patients can be found in Table 2. As expected, gain-of-function mutations in *STAT1* were the most common identified defects (Fig. 4). Specifically, 15 CMC patients were shown to carry heterozygous missense mutations in *STAT1*; some of these patients have been previously published [22, 29]. Out of these, one patient additionally had a compound heterozygous *CARD9* mutation and another one a mutation in *IL17RA*.

**Table 2:** Detected mutations in CMC patients: summary of detected disease-causing variants and clinical presentation of patients. Hom, homozygous; het, heterozygous; hemi, hemizygous

Patient	From	NIH HIES score	Age at first clin. Presentation (years)	Age at genet. diagn. (years)	Gene	Base exchange	AA exchange	Zygosity	Variant published? (ref.) functional validation?	Clinical presentation
CMC01	Germany	n.a	n.a	4	*AIRE*	c.1411C > T	p.R471C	het	Yes [56] No	Recurrent oral candida infections in infancy, recurrent respiratory tract infections, obstructive bronchitis, respectively asthmatic symptoms, antibodies to IL28A and IL28B
CMC02	Turkey	12	3	26	*CARD9*	c.883C > T	p.Q295*	hom	Yes [15] Yes	Candida meningoencephalitis, a case report was published following successful treatment with G-CSF [57]. Consanguinity. His twin brother with the same mutation had only mild chronic mucocutaneous candidiasis without invasive fungal infections
CMC03	Turkey	8	7	21	*CARD9*	c.1118G > C	p.R373P	hom	Yes [44] Yes	Candida meningoencephalitis, granulomatous fungal infection of lymph node (biopsy revealed candida), liver cirrhosis with portal hypertension (liver biopsy showed focal microabscesses including candida hyphae and spores), hepatosplenomegaly, *M. furfur* skin infection. Bone marrow biopsy w hypercellular marrow and increased plasma cells
CMC04	Turkey	n.a	n.a	n.a	*CARD9*	c.586A > G	p.K196E	hom	Yes [58] Yes	Chronic mucocutaneous candidiasis, two siblings also affected with same mutation
CMC05	Germany	n.a	< 1	31	CARD9*	c.1492_1493delAG	p.S498Ffs*2	het	No	Chronic mucocutaneous candidiasis of oral cavity, nails and genitourinary tract, abscesses and recurrent aphthous and ulcerous lesions, clinical improvement upon fluconazole prophylaxis. CD4 lymphopenia, recurrent episodes of panlymphopenia, mild antibody deficiency. A case report on the clinical presentation of this patient was published in 2002 before the present genetic testing [29]. *Also has *STAT1* mutation
						c.1434 + 1G > C	IVS11 + 1(C > G)	het	Yes [59] Yes	
CMC21	Germany	n.a	n.a	11	*IL12RB1*	c.1791 + 2 T > G	IVS16 + 2 (A > C)	hom	Yes [60] Yes	Mycobacterial disease
CMC06	Turkey	5	9	10	*IL17RA*	c.164_165insTACC	p.C57Yfs*5	hom	Yes [61] No	Prolonged and resistant oral and superficial candidiasis. Consanguinity. One affected brother with same mutation
CMC07	Germany	6	8	21	IL17RA*	c.196C > T	p.R66*	het	Yes [61] No	Chronic mucocutaneous candidiasis with recurrent fungal infections including recurrent oral candida infections, esophagitis and nail infections since childhood as well as two episodes of skin abscesses. Ulcerative esophageal lesions. Father also had recurrent candida infections, passed away due to esophageal carcinoma. *Also has *STAT1* mutation
					IL17RA*	c.958 T > C	p.W320R	het	Yes [62] No	
CMC08	USA	n.a	n.a	10	*STAT1*	c.1154C > A	p.T385K	het	Yes [22] Yes	Recurrent thrush, esophageal candidiasis, alopecia, hypothyroidism, failure to thrive, history of pneumonia, underwent hematopoietic stem cell transplantation
CMC09	Germany	n.a	n.a	19	*STAT1*	c.596 T > C	p.L199P	het	No	No clinical data available
CMC05	Germany	n.a	< 1	31	STAT1*	c.865 T > A	p.Y289N	het	No	Chronic mucocutaneous candidiasis of oral cavity, nails and genitourinary tract, abscesses and recurrent aphthous and ulcerous lesions, clinical improvement upon fluconazole prophylaxis. CD4 lymphopenia, recurrent episodes of panlymphopenia, mild antibody deficiency. A case report on the clinical presentation of this patient was published in 2002 before the present genetic testing [29]. *Also has CARD9 mutation
CMC10	Germany	7	1	40	*STAT1*	c.821G > A	p.R274Q	het	Yes [14] Yes	Recurrent candida esophagitis with proof of *C. albicans* with multiple drug resistance, status post esophageal rupture, IgA deficiency
CMC11	France	n.a	n.a	44	*STAT1*	c.970 T > C	p.C324R	het	Yes [63] Yes	Chronic mucocutaneous candidiasis with autoimmune features, 4 children also affected
CMC12	Germany	17	43	50	*STAT1*	c.1175 T > C	p.M392T	het	Yes [64] Yes	Candida esophagitis with inflammatory esophageal stenosis, history of recurrent fungal skin infections, onychomycosis, recurrent pneumonias with bronchiectasis
CMC13	Algeria	n.a	< 1	n.a	*STAT1*	c.1154C > T	p.T385M	het	Yes [65] Yes	Oral candidiasis from the age of 7 months, onychomycosis, pulmonary candida infection, failure to thrive
CMC14	Great Britain	n.a	n.a	n.a	*STAT1*	c.800C > T	p.A267V	het	Yes [13] Yes	CMC, father and sister also affected
CMC15	Great Britain	n.a	n.a	n.a	*STAT1*	c.800C > T	p.A267V	het	Yes [13] Yes	CMC, father, two siblings and child also affected
CMC16	Great Britain	n.a	n.a	n.a	*STAT1*	c.820C > T	p.R274W	het	Yes [13] Yes	CMC, hypothyroidism, autoimmune hepatitis, mother and one sibling also affected
CMC17	Great Britain	n.a	n.a	n.a	*STAT1*	c.820C > T	p.R274W	het	Yes [13] Yes	CMC, grandmother and mother also affected
CMC07	Germany	6	8	21	STAT1*	c.846A > C	p.E282D	het	No	Chronic mucocutaneous candidiasis with recurrent fungal infections including recurrent oral candida infections, esophagitis and nail infections since childhood as well as two episodes of skin abscesses. Ulcerative esophageal lesions. Father also had recurrent candida infections, passed away due to esophageal carcinoma. *Also has *IL17RA* mutation
CMC18	Israel	n.a	3	26	*STAT1*	c.1310C > T	p.T437I	het	Yes [66] Yes	Oral ulcers, esophageal ulcers, oral thrush, esophageal candidiasis, cutaneous candidiasis
CMC22	Germany	22	16	20	*STAT1*	c.1154C > T	p.T385M	het	Yes [65] Yes	Recurrent pneumonias with bronchiectasis and status post left lower lobe resection aged 12 years, candida esophagitis, disseminated non-tuberculous mycobacteria infection (lung, ascites, bone marrow), autoimmune thyroiditis, pancytopenia, immunoglobulin subclass deficiency with lack of memory B cells, passed away due to hepatopulmonary syndrome
CMC23	Germany	n.a	n.a	16	*STAT1*	c.1024A > C	p.T342P	het	No	Chronic mucocutaneous candidiasis
CMC19	Great Britain	15	1	10	*STAT3*	c.1708G > A	p.D570N	het	Yes [67] Yes	Mucocutaneous candidiasis from first year of life on with recurrent infection of nails, skin and scalp, Crohn’s disease necessitating immunosuppressive treatment
CMC20	Great Britain	30	n.a	47	*STAT3*	c.1680_1682delCTT	p.F561del	het	Yes [32] Yes	Chronic mucocutaneous candidiasis with deep and disseminated dermatophyte infection. Eczema, recurrent skin abscesses, elevated IgE and eosinophilia. A case report on this patient was published [32]
CMC24	Great Britain	13	29	34	*STAT3*	c.1708G > A	p.D570N	het	Yes [67] Yes	Recurrent candidiasis of oral cavity, skin and nails since childhood, one episode of fungal infection of the lung confirmed by bronchoscopy. Multiple bacterial pulmonary infections with at least one pneumonia as well as a pulmonary abscess requiring surgery, recurrent warts. Significant mental retardation. Eosinophils and IgE within normal range

Four CMC patients were found to have homozygous or compound heterozygous mutations in *CARD9*. One patient had a homozygous nonsense mutation, two had homozygous missense mutations, and one patient had a compound heterozygous mutation consisting of a heterozygous frameshift mutation and a heterozygous splice site mutation. Two of these four patients had suffered from candida meningoencephalitis.

**Fig. 5:**
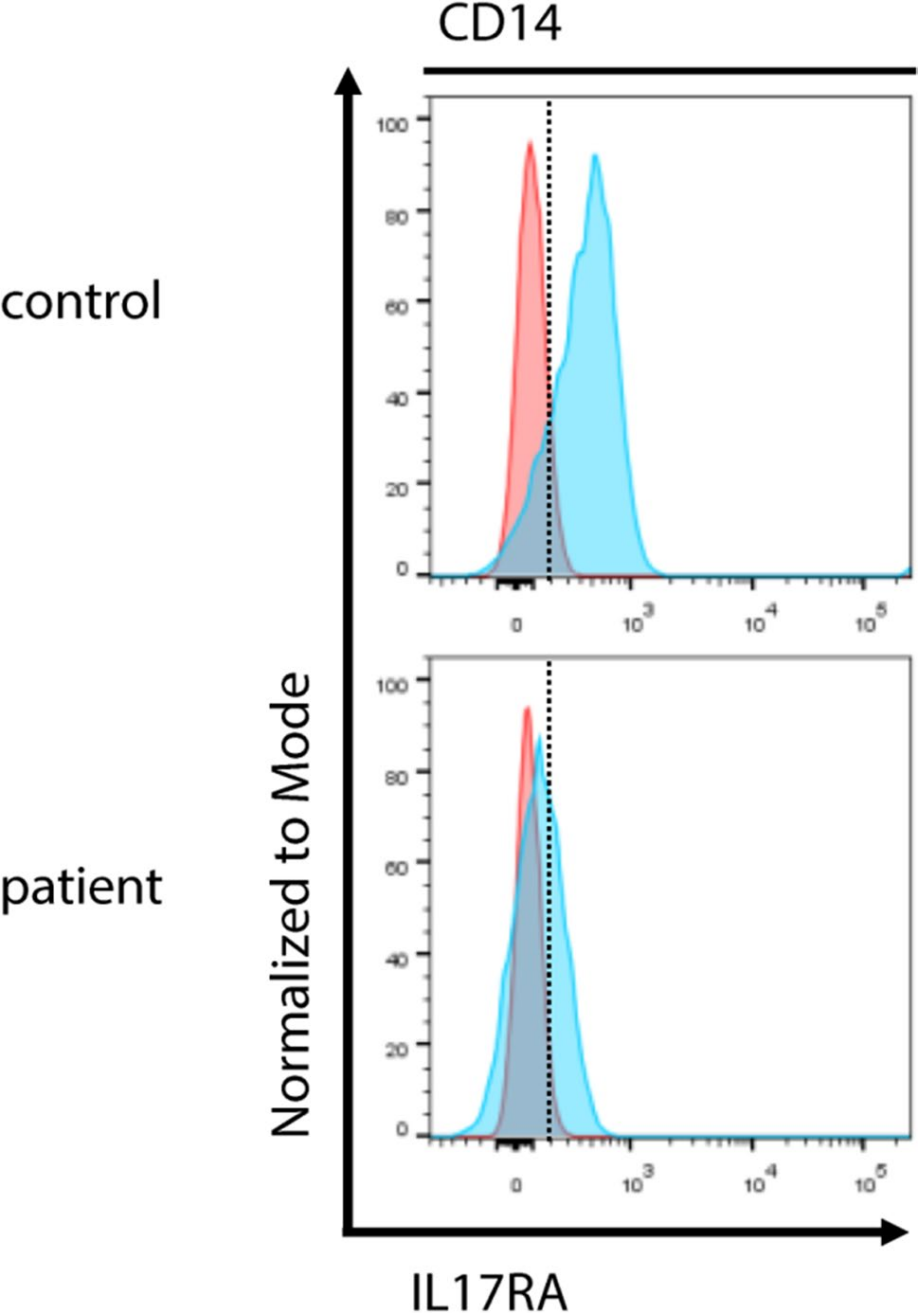
Assessment of *IL17RA* expression: flow cytometric analysis of *IL17RA* in CD14^+^ cells shows reduced surface expression in patient CMC06 with the homozygous frameshift variant p.C57Yfs*5

Furthermore, two patients were found to have mutations in *IL17RA*. One patient had a homozygous frameshift mutation, where flow cytometric analysis showed severely reduced surface expression of *IL17RA* (see Fig. 5). The other patient had a heterozygous nonsense mutation (p.R66*) and a heterozygous missense variant predicted to be damaging (p.W320R). Primary cells of this patient for staining were not available.

One patient was shown to have a heterozygous missense variant in *AIRE (*p.R471C), which has previously been published as being associated with autoimmune features but not overt APECED (autoimmune-polyendocrinopathy-candidiasis-ectodermal dystrophy). He had suffered from recurrent oral candida infections in infancy and was reported to have recurrent respiratory tract infections and obstructive bronchitis. Anticytokine autoantibodies against IL17 or IL22, which are commonly observed in APECED, were below the detection level. However, the patient was shown to have autoantibodies against IL28A/B, which have previously been described in APECED, but do not belong to the most prevalent anticytokine autoantibodies [30]. The type III interferons IL28A/B have been shown to play a role in antifungal immunity [31], thus these autoantibodies may play a role in the development of CMC.

Finally, three patients with the clinical diagnosis of CMC had heterozygous mutations located in the linker domain of *STAT3* (p.F561del and p.D570N). Functional workup of the F561del mutation had shown normal *STAT3* expression and functional phosphorylation of *STAT1* and *STAT3*. Further analysis showed reduced *STAT3* target gene activation and increased *STAT1* target gene activation, thus mimicking a *STAT1* gain-of-function phenotype [32] with a clinical presentation of mucocutaneous candidiasis.

### Identified Genetic Defects in the Previously Sanger-Sequenced Subcohort

Out of 90 patients, in whom previous Sanger sequencing PCR amplification of the suspected underlying target gene (*STAT3*, *DOCK8*, *PGM3*, *STAT1*) had not led to a definite molecular diagnosis, we were only able to detect a genetic defect in 13 patients, i.e., 14.4% by panel sequencing. Here, the most common defects identified by panel sequencing were *STAT3* mutations (7 patients), followed by homozygous *DOCK8* variants (3 patients). One patient each had a homozygous or compound heterozygous mutation in *CARD9*, *ZNF341*, and *AIRE*. Out of the seven patients with *STAT3* mutations, three had previously undergone Sanger sequencing for *STAT3*. This suggests that although Sanger sequencing was considered the gold standard for genetic testing, this method is not free of errors itself and in the case of strong clinical suspicion, further genetic testing should be considered.

## Discussion

In this study, we used a targeted gene panel sequencing approach to search for underlying mutations in a cohort of 211 patients clinically diagnosed with HIES and 64 patients diagnosed with CMC by their referring physicians. In total, we have detected 87 mutations in 78 patients. This translates into a diagnostic hit rate of 28.4% in our cohort, which is in line with other studies published on next-generation sequencing approaches in primary immunodeficiencies, which have reported rates of 15 to 40% [33–36], depending on patient preselection. Out of the 87 detected mutations, 32 constituted novel mutations, whereas 55 mutations had been published before.

Due to many patients within this cohort hailing from Middle Eastern countries, such as Egypt, Turkey, Iran, and Morocco, where consanguinity is more common, there was a selection bias, which may account for the fact that *DOCK8* mutations were the most commonly identified defects in this cohort. Previous reports have identified STAT defects as the most common defects in European cohorts [37]. Similarly, among patients with European ancestry, also in this cohort, *STAT1* and *STAT3* defects were by far the most prevalent. Furthermore, we cannot rule out that previous Sanger sequencing of a part of the cohort may have led to further bias. Out of an overall cohort of 613 HIES and 93 CMC patients analyzed by our laboratory, 322 had undergone Sanger sequencing for the target gene deemed most likely, revealing 91 *STAT3*, 39 *DOCK8*, and 5 *PGM3* mutations in HIES and 23 *STAT1* and 4 *CARD9* mutations in the CMC cohort. Thus, overall, *STAT3* and *STAT1* constituted the most frequent defects.

While it was originally published that *TYK2* mutations may cause a hyper-IgE phenotype, this phenotype could not be replicated and hence *TYK2* has been removed from the list of HIES defects in the IUIS classification. Also, in our cohort, we did not detect any mutations in *TYK2*, confirming that an HIES phenotype is not typically caused by mutations in *TYK2*.

The three most commonly reported clinical features in the described cohort were IgE elevation, eczema, and eosinophilia, which are common to all hyper-IgE phenotypes but are unfortunately largely unspecific. The most common condition with this phenotype is severe atopic dermatitis [38]. Several authors have addressed the issue of how to distinguish primary immunodeficiency from atopic disease [38–40]. Schimke et al. found internal abscesses and severe infections among other features predictive of primary immunodeficiency (PID) [38]. In this cohort, 8.2% of patients had no history of major or increased minor infections, whereas 41.7% did not have a history of severe infections (pneumonia or other severe or fatal infection), rendering the diagnosis of immunodeficiency uncertain. Thus, despite their clinical diagnosis of HIES, some patients may in fact suffer from other conditions within the spectrum of hyper-IgE phenotypes. This hypothesis is supported by the fact that we have detected variants in filaggrin (*FLG*) in patients from this cohort, some of which are known to be associated with ichthyosis vulgaris and atopic dermatitis.

In addition, individual noninfectious features were nonspecific to the mutational status. A characteristic facies was the most commonly reported skeletal feature in this cohort (23.1%); however, this is not an objectively quantifiable criterion and relies heavily on the expertise of the referring physician. Bone fractures with inadequate trauma were reported in six patients with *STAT3* mutations (6/21) but also in six patients without detected genetic defect (6/197). Severe scoliosis was reported in two patients with *STAT3* defects (2/21) and seven patients without detected genetic defects (7/197). Pneumatocele formation was reported in five patients (5/21) with *STAT3* mutations, one (1/3) with *AIRE* mutation, and eight (8/197) without any detected genetic defect. This is consistent with earlier findings from Schimke et al. describing pneumatoceles, nail or mucocutaneous candidiasis, bone fractures without adequate trauma, and scoliosis to be predictive of *STAT3*. While, thus, distinguishing *STAT3* from other genetic defects, these features fail to clearly distinguish patients with and without a genetic defect. These findings highlight again that each individual clinical feature by itself is nonspecific. For this reason, scores such as the HIES NIH score were developed to predict underlying genetic defects with a higher likelihood. However, these are only available for the more common and established hyper-IgE phenotypes such as *STAT3* and *DOCK8*, and an overall score to our knowledge so far does not exist. Furthermore, scoring systems such as the NIH HIES score may impede an early diagnosis as they rely on morphological criteria that develop late in childhood (e.g., teeth, skeletal anomalies) and on the manifestation of infections and end-organ damage, which may take years to become obvious. This issue may be circumvented by early genetic analysis for underlying defects. Broader sequencing may help particularly to identify atypical presentations.

In this study, the targeted panel sequencing approach has allowed us to identify mutations in patients with atypical clinical presentations, such as *CARD9* mutations in patients with a clinical diagnosis of the hyper-IgE syndrome, *STAT3* mutations in patients with chronic mucocutaneous candidiasis and patients with low HIES scores that may otherwise have been missed, or a *WAS* mutation in a patient with a typical HIES phenotype.

This highlights the importance of making a genetic diagnosis, as the clinical picture may be variable and the genetic background unexpected. However, the latter may have crucial relevance regarding the counseling and treatment of the patient and their family members. Furthermore, several patients with mutations in multiple genes were identified, which may contribute to atypical, complex, or more severe phenotypes. In addition, the in-depth molecular characterization of underlying mutations and the involved pathways constitutes a prerequisite for the identification of novel therapeutic targets and the development of targeted drug therapy and novel therapeutic approaches such as gene surgery.

In our experience, the targeted panel sequencing approach provided a cost-effective first-line genetic screening method. Material costs for panel sequencing amounted to €141 per sample, whereas commercial whole exome sequencing costs €600–€700 including data processing for a single patient and approximately €1400 for a trio of child and parents. Data analysis for panel sequencing does not require complex bioinformatical analysis but can be performed on preexisting software, rendering analysis time and cost effective.

Covering a smaller number of genes highly preselected for those known to be disease causing reduces the effort and time spent on interpreting variants of unclear significance. While panel sequencing precludes the identification of novel genetic defects, the process is more time efficient. Particularly, in a diagnostic setting, fast turnaround times are crucial to ensure a quick diagnosis, thus favoring a panel sequencing approach.

The average coverage of detected mutations in this study amounted to 914 reads. The achieved high coverage is one of the distinct advantages of panel sequencing over the whole exome and especially whole genome sequencing and is crucial to reduce errors [41, 42], particularly in a diagnostic setting. Disadvantages to panel sequencing include the constant need to update the panel to reflect the current literature and the need for further follow-up, including further sequencing in case no variants are detected. This is also a limitation of this study as the majority of patients without detection of a genetic defect were not subjected to further genetic workup. The variable number of genes sequenced due to the continuous adaptation of the panel may be regarded as a further limitation of this study.

For the time being, however, we propose a stepwise approach with targeted panel sequencing as the first step in genetic diagnostics. For patients, in whom no mutation could be detected, further workup, including whole exome sequencing, should be considered especially in the case of young age and positive family history and if further family members are available for sequencing. In the future, whole exome sequencing (WES) will probably substitute panel sequencing in the diagnostic setting as both WES and whole genome sequencing and the ensuing data analysis are becoming cheaper and quicker and databases with annotation of variants are improving. The continued improvement of sequencing technologies and bioinformatics analysis will further facilitate the discovery of single nucleotide variants and indels (insertions/deletions) and other structural rearrangements in the future.

In conclusion, we present a targeted panel sequencing approach for the identification of genetic variants underlying the clinical spectrum of hyper-IgE syndromes and mucocutaneous candidiasis. In total, we have detected 87 mutations in 78 out of 275 patients, of which 32 were novel mutations.

## Supplementary Information

Below is the link to the electronic supplementary material.

Supplementary file1 (PDF 110 KB)

## Data Availability

Data can be made available upon request.
