# Supplementary material for: Genetic Analysis of a Cohort of 275 Patients with Hyper-IgE Syndromes and/or Chronic Mucocutaneous Candidiasis
Source: J Clin Immunol. 2021 Aug 14;41(8):1804–38. doi: 10.1007/s10875-021-01086-4 (PMC8604890; doi:10.1007/s10875-021-01086-4)
Supplement: Supplementary file 1 — Supplementary file1 (PDF 110 KB) [file 10875_2021_1086_MOESM1_ESM.pdf]

Supplemental Table 1: Sequenced genes: The gene panel was updated regularly to optimize coverage and include new candidate genes starting from 15 published genes (CMC_HIES 1.0).

CMC_HIES 1.0	CMC_HIES 3.0	CCI PID 2.0
AIRE	AHR	AICDA
CARD9	AIRE	AIRE
CYBA	CARD9	BBX
CYBB	CLEC7A	BTK
DOCK8	CYBA	CARD11
IL17F	CYBB	CARD9
IL17R	DOCK8	CD274
IRF8	ICAM1	CD28
PGM3	IL12A	CECR1
SPINK5	IL12RB1	CR2
STAT1	IL17A	CTLA4
STAT3	IL17F	DKC1
STK4	IL17RA	DOCK8
TYK2	IL17RB	FCHO1
	IL22	FOXP3
	IL6	ICOS
	IL6ST	IKZF1
	IRAK4	IL10RA
	IRF8	IL10RB
	MYO5B	IL17A
	PGM3	IL17RA
	SPINK5	ITSN2
	STAT1	LRBA
	STAT3	MICALL2
	STK4	MYH9
	SYK	MYO5B
	TRAF3IP2	NCF2
	TYK2	NFKB1
		NFKB2
		NFKBIA
		NOD2
		P2RX7
		PDCD1
		PGM3
		PIK3C2A
		PIK3CD
		PIK3R1
		PIK3R4
		PTEN
		RAG1
		RAG2
		REL
		RELA
		RELB
		RLTPR
		SEC61A1
		SH2D1A
		SPINK5
		STAT1
		STAT3
		STXBP2
		TNFRSF13B
		TNFRSF13C
		TNFRSF17
		TNFSF10
		TNFSF13
		TNFSF13B
		TYK2
		WAS
		XIAP
		ZNF341
